# Intermittent fasting versus continuous energy restriction in MASLD: a systematic review and meta-analysis

**DOI:** 10.3389/fnut.2026.1833688

**Published:** 2026-05-13

**Authors:** Hanlu Li, Tianju Li

**Affiliations:** Department of Infectious Diseases, Beibei Affiliated Hospital of Chongqing Medical University, Chongqing, China

**Keywords:** continuous energy restriction, intermittent fasting, meta-analysis, metabolic dysfunction-associated steatotic liver disease, randomized controlled trials

## Abstract

**Background:**

Intermittent fasting (IF) and continuous energy restriction (CER) are widely used dietary approaches for metabolic dysfunction–associated steatotic liver disease (MASLD), yet their comparative efficacy remains uncertain.

**Methods:**

PubMed, Embase, Web of Science, and Cochrane Central were searched from inception to August 1, 2025, for randomized controlled trials comparing intermittent fasting (IF) with continuous energy restriction (CER) in adults with NAFLD, MAFLD, or MASLD. Risk of bias was assessed using RoB 2, and weighted mean differences were pooled using random-effects models.

**Results:**

Nine trials (*n* = 894) were included, involving time-restricted feeding (TRF), alternate-day fasting (ADF), and 5:2 fasting (whole-day fasting, WDF). Compared with CER, IF was associated with greater reductions in body weight (MD = −1.29 kg; 95% CI, −1.98 to −0.61), BMI (MD = −0.34 kg/m^2^; 95% CI, −0.55 to −0.13), LDL-C (MD = −0.08 mmol/L; 95% CI, −0.15 to −0.01), and controlled attenuation parameter (CAP) (MD = −15.13 dB/m; 95% CI, −28.87 to −1.39). No significant between-group differences were observed for magnetic resonance imaging–proton density fat fraction (MRI-PDFF), liver stiffness, transaminases, glucose-related indices, total cholesterol, triglycerides, or HDL-C. In subgroup analyses, WDF was associated with a greater reduction in CAP, whereas TRF appeared to be associated with greater reductions in body weight and BMI.

**Conclusion:**

In patients with MASLD, intermittent fasting was associated with greater reductions in body weight, BMI, and LDL-C than continuous energy restriction. Although CAP-based analyses suggested a possible advantage of intermittent fasting for hepatic steatosis, this finding was not confirmed by MRI-PDFF and was no longer statistically significant in sensitivity analyses. Further large-scale, long-term randomized trials are needed to confirm these findings.

**Systematic review registration:**

https://www.crd.york.ac.uk/PROSPERO/view/CRD420251149184, Identifier: CRD420251149184.

## Introduction

Metabolic dysfunction–associated steatotic liver disease (MASLD), previously known as nonalcoholic fatty liver disease (NAFLD), is among the most prevalent chronic liver diseases worldwide. MASLD is defined as hepatic steatosis in the presence of at least one cardiometabolic risk factor and in the absence of excessive alcohol intake or other dominant causes of steatosis. This term was introduced in the 2023 multisociety consensus to replace NAFLD (1). The global prevalence of MASLD has increased from 25.3 to 38.2%, indicating nearly a 50% rise over the past three decades (2). The development of MASLD is strongly associated with obesity, insulin resistance, type 2 diabetes mellitus, and metabolic syndrome. A subset of patients may progress to metabolic dysfunction–associated steatohepatitis (MASH), liver fibrosis, cirrhosis, or hepatocellular carcinoma, consequently increasing the risk of liver-related and cardiovascular mortality (1, 3, 4). In this study, the term MASLD is used throughout the manuscript in accordance with current consensus nomenclature. The terms NAFLD and MAFLD are retained only where appropriate, such as in the description of prior studies or diagnostic criteria.

Although pharmacological treatment options for MASLD/MASH have advanced, lifestyle intervention remains the cornerstone of management, and approved disease-specific therapies are still limited to selected patient populations (5, 6). As MASLD mainly affects overweight or obese individuals and is strongly associated with cardiovascular risk, lifestyle modification—particularly dietary and physical activity interventions—plays a central role in disease management (4, 5). Evidence suggests that a 7–10% weight reduction can significantly alleviate hepatic steatosis and inflammation (7). Continuous energy restriction is a conventional dietary approach for weight loss and can improve hepatic steatosis and metabolic risk factors in patients with NAFLD/MASLD. Meanwhile, randomized clinical studies have suggested that intermittent calorie restriction may improve liver-related outcomes in patients with NAFLD, further supporting the potential role of fasting-based dietary strategies in this population (8). However, maintaining long-term adherence is challenging, as compliance often declines over time (9). Recently, intermittent fasting (IF) has attracted growing attention as a potentially more sustainable approach to managing obesity and metabolic disorders. IF may provide benefits comparable to those of continuous energy restriction (CER) in body weight, glycemic control, and cardiometabolic health, and may be more feasible for certain populations (10). The basic principle of IF involves abstaining from caloric intake during specific daily or weekly periods while permitting ad libitum intake during the remaining time (11). The health effects of intermittent fasting may result not only from reduced energy intake but also from metabolic switching between fed and fasting states (12).

Common IF regimens include time-restricted feeding (TRF), alternate-day fasting (ADF), and 5:2 fasting, also referred to as whole-day fasting (WDF). TRF limits daily caloric intake to a predetermined eating window, typically less than 12 h (13). ADF alternates fasting or calorie-restricted days with ad libitum feeding days, whereas WDF restricts caloric intake on two nonconsecutive days per week while permitting unrestricted eating on the remaining 5 days (11, 13, 14). Previous meta-analyses have suggested that IF may improve hepatic outcomes in patients with NAFLD (15). More recently, randomized controlled trials have directly compared intermittent and continuous energy restriction strategies in patients with steatotic liver disease. For example, Sun et al. reported a randomized clinical trial comparing intermittent versus continuous calorie restriction in patients with MASLD, further expanding the disease-specific evidence base (16). In addition, a recent systematic review and network meta-analysis suggested that intermittent fasting regimens may improve hepatic and metabolic outcomes in MASLD, although the comparative efficacy across fasting regimens and against CER remains uncertain (17). In this context, we conducted a systematic review and meta-analysis to compare the effects of IF and CER on hepatic and metabolic outcomes in patients with MASLD, with the goal of providing robust evidence to inform clinical dietary management strategies.

## Methods

### Study design and registration

This systematic review and meta-analysis followed the Preferred Reporting Items for Systematic Reviews and Meta-Analyses (PRISMA) guidelines and was prospectively registered in the International Prospective Register of Systematic Reviews (PROSPERO; ID: CRD420251149184).

### Search strategy

A comprehensive literature search was conducted in Cochrane Central, Web of Science, PubMed, and Embase from inception to August 1, 2025 to identify eligible studies. The search strategy combined controlled vocabulary terms (e.g., MeSH and Emtree terms, where applicable) and free-text keywords related to NAFLD, MAFLD, MASLD, intermittent fasting, time-restricted feeding, alternate-day fasting, 5:2 fasting, whole-day fasting, calorie restriction, and energy restriction. The complete search strategies for all databases are provided in Supplementary Table S1. No restrictions were imposed regarding language, publication date, or publication status.

### Study selection

After removing duplicates, two reviewers (HLL and TJL) independently screened titles and abstracts to identify studies that met the inclusion criteria. Full-text articles of potentially relevant studies were subsequently retrieved and independently assessed by the same reviewers. Any discrepancies were resolved through discussion until consensus was achieved.

### Inclusion and exclusion criteria

To ensure methodological rigor, the eligibility criteria were defined according to the Population, Intervention, Comparison, Outcomes, and Study design (PICOS) framework. Population: Adults (≥18 years) diagnosed with NAFLD, MAFLD, or MASLD according to study-defined diagnostic criteria, including imaging-based methods such as ultrasound, controlled attenuation parameter (CAP), or magnetic resonance imaging–proton density fat fraction (MRI-PDFF). Intervention: IF regimens, including TRF, ADF, or WDF. Comparison: CER as the control condition. Outcomes: At least one relevant hepatic or metabolic outcome had to be reported, including hepatic steatosis/fibrosis parameters, anthropometric measures, lipid profile, or glucose-related indices. Study design: Only randomized controlled trials (RCTs), including both parallel-group and crossover designs, were eligible for inclusion.

The exclusion criteria were as follows: (1) animal studies; (2) studies involving individuals younger than 18 years or pregnant women; (3) non-randomized studies; (4) studies without an eligible IF intervention; (5) studies without a CER comparator; (6) studies conducted in populations other than fatty liver disease; (7) conference abstracts; and (8) studies that did not provide sufficient quantitative data for meta-analysis.

### Data extraction

Two reviewers independently extracted data from each included study using a standardized data collection form. Any discrepancies were resolved through discussion until consensus was reached. Extracted information included the first author’s name, publication year, study design, dietary guidance, diagnostic method, number of study arms, sample size in each group, intervention duration, details of the IF protocol and CER comparator, participant characteristics (including age and BMI), and reported hepatic and metabolic outcomes. For quantitative synthesis, baseline and post-intervention outcome data were extracted, including means, standard deviations, medians, and 95% confidence intervals, as available. When studies reported change-from-baseline values, these were preferentially extracted. For crossover trials, information relevant to within-participant comparisons was extracted whenever available. For trials with multiple intervention arms, only eligible arms that were comparable with the CER control group were included. Study arms involving additional co-interventions (e.g., increased physical activity, behavioral support, or nutrient-enriched diets) that were not applied equally across comparison groups were excluded to maintain comparability.

### Risk of bias assessment

The methodological quality of included RCTs was assessed using the Revised Cochrane Risk-of-Bias Tool for Randomized Trials (RoB 2), which evaluates five domains: (1) randomization process, (2) deviations from intended interventions, (3) missing outcome data, (4) outcome measurement, and (5) selective reporting.

Each domain was rated as having a “low risk,” “some concerns,” or “high risk” of bias, and an overall risk-of-bias judgment was derived accordingly. Blinding of participants and personnel was generally infeasible due to the nature of dietary interventions; however, blinding of outcome assessors (e.g., radiologists or laboratory technicians) was encouraged and considered a key indicator of study quality.

Overall study quality was classified as low risk (all domains rated “low”), unclear risk (one or more domains rated “some concerns”), or high risk (one or more domains rated “high”).

### Statistical analysis

For all continuous outcomes, effect sizes were calculated as the difference in mean change from baseline to post-intervention between the IF and CER groups. When studies reported multiple follow-up time points, data from the end of the intervention or the end of the supervised intervention phase were prioritized. For crossover trials, paired within-participant comparisons were used whenever such data were available. When paired effect estimates were unavailable, the most appropriate summary data were used, and the influence of these studies was further examined in sensitivity analyses excluding crossover trials. A random-effects model was applied to pool mean differences (MDs) with corresponding 95% confidence intervals (CIs), and the results were presented using forest plots. Between-study heterogeneity was quantified using the I^2^ statistic, with I^2^ > 50% considered substantial heterogeneity. To explore potential sources of heterogeneity, prespecified subgroup analyses were conducted according to IF regimen (TRF, ADF, or WDF), intervention duration (<13 vs. >13 weeks), presence of between-group differences in energy intake, BMI (<30 vs. ≥30 kg/m^2^), mean age (≤43 vs. >43 years), ethnicity (Asian vs. Caucasian), and imaging modality (magnetic resonance vs. ultrasound), where applicable. Sensitivity analyses were performed by excluding crossover trials and studies with sample sizes < 50 to evaluate the robustness of the pooled estimates. When sufficient studies were available, publication bias was assessed by visual inspection of funnel plots and Egger’s regression test. A two-sided *p* value < 0.05 was considered statistically significant. All statistical analyses were conducted using R software (version 4.5.1). The certainty of evidence for each outcome was assessed using the Grading of Recommendations Assessment, Development and Evaluation (GRADE) approach, and the corresponding ratings are presented in Supplementary Table S9.

## Results

**Figure 1:**
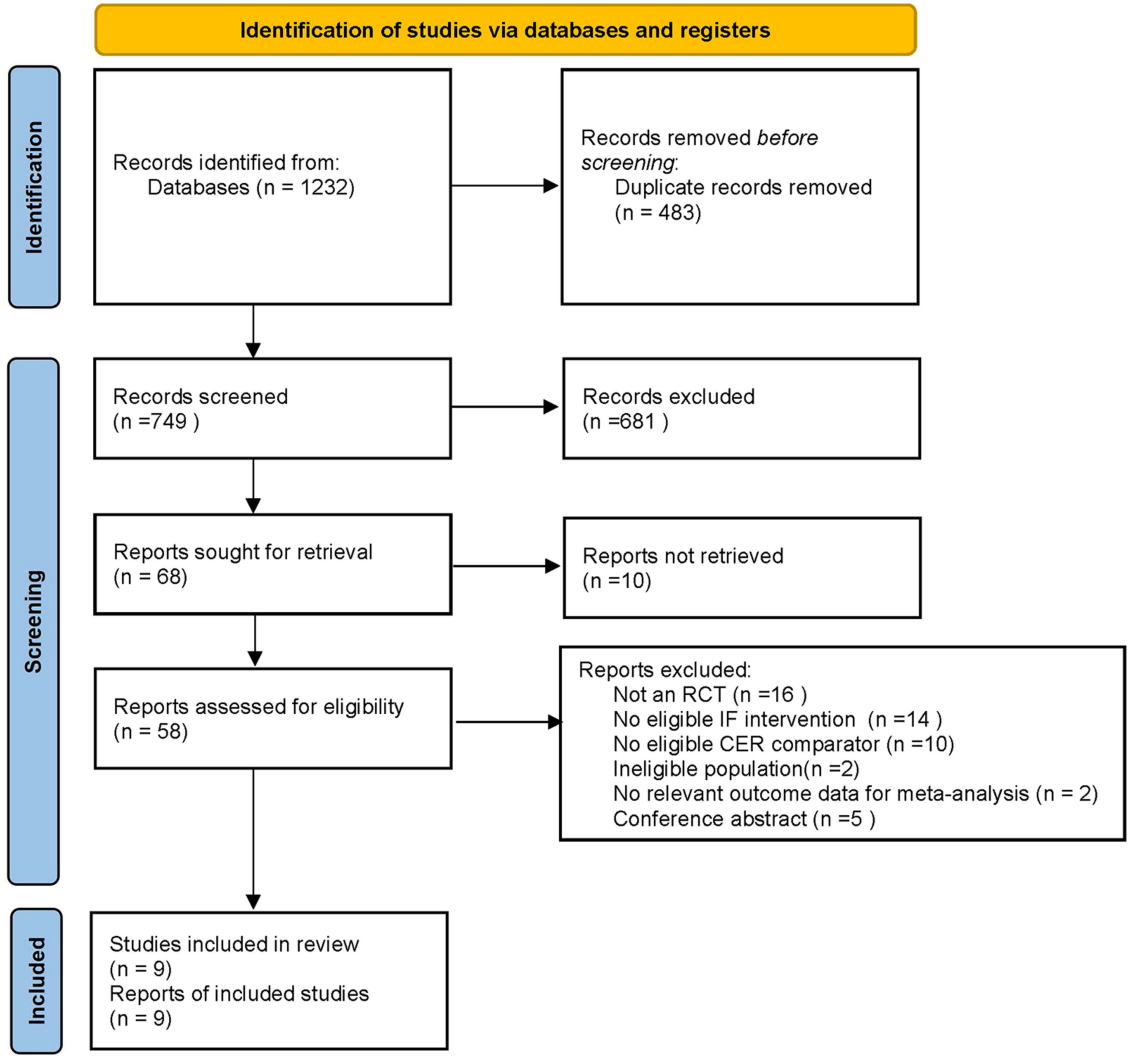
PRISMA flow diagram of study selection for the systematic review and meta-analysis.

A total of 1,232 records were initially retrieved from database searches. After removing 483 duplicates, 749 records remained for title and abstract screening. Of these, 681 were excluded for not meeting the inclusion criteria, leaving 68 full-text articles for detailed assessment. After full-text assessment, nine randomized controlled trials (RCTs) met the eligibility criteria and were included in the meta-analysis (Figure 1).

### Study characteristics

The characteristics of the nine included RCTs are summarized in Table 1. Eight trials employed a parallel-group design (16, 18–24), and one utilized a crossover design (25). The studies published between 2019 and 2025, included 508 participants in the IF groups and 386 in the CER groups. All trials assessed hepatic steatosis using ultrasound or MRI-PDFF. Participants’ mean age ranged from 31 to 61 years, and median BMI ranged from 26 to 33 kg/m^2^. Both male and female participants were included. Diagnoses were primarily based on NAFLD, MAFLD, or MASLD criteria. Sample sizes ranged from 15 to 110, and intervention durations ranged from 8 to 52 weeks. Across the eligible intervention arms, six used TRF, one used ADF, and three used WDF. Four studies reported changes in energy intake from baseline (16, 18, 23, 25), two provided both baseline and end-of-intervention values (22, 25), and three reported mean energy intake across the intervention period (19, 21, 24). Seven trials found no significant between-group differences in energy intake during the intervention (21, 24, 25), in change from baseline (16, 18, 25), or in post-intervention energy intake (20, 22), whereas two studies reported lower caloric intake in the IF groups than in the CER groups (23, 24).

**Table 1:** Characteristics of the included randomized controlled trials.

Reference	Population	Imaging technique	Intervention		Duration	Sample size		Age (year)		BMI (kg/m^2^)	
			IF	CER		IF	CER			IF	CER
TRF											
Oh et al. (18)	Overweight or obese and diagnosed with MASLD	Ultrasound or magnetic resonance	Food intake (8 h): 500 kcal/d energy deficit Fasting period (16 h): 0 kcal	500 kcal/d energy deficit	16 weeks, at least two contacts per week	*N* = 110 started *N* = 78 finished (70.9%)	*N* = 110 started *N* = 89 finished (80.9%)	42 ± 10	44 ± 9	29 ± 5	28 ± 3
Karimi et al. (19)	MAFLD	Ultrasound	Food intake (8 h): ad libitum intake, Fasting period (16 h): 0 kcal	300 kcal/d energy deficit	3-month, Weekly calls	*N* = 26 started *N* = 20 finished (76.92%)	*N* = 26 started *N* = 22 finished (92.3%)	41 ± 6	40 ± 8	31 ± 3	30 ± 4
Karahan et al. (20)	MAFLD	Ultrasound	Food intake (8 h): 22-25 kcal/kg, Fasting period (16 h): 0 kcal	22-25 kcal/kg	8 weeks, Fortnightly contacts.	*N* = 26, Compliance was not reported	*N* = 22, Compliance was not reported	NA	NA	32 (29–35)	33 (30–37)
Feehan et al. (25)	NAFLD	Ultrasound	Food intake (8 h): ad libitum intake, Fasting period (16 h): 0 kcal	Information and advice on healthy lifestyle (low-calorie and low-fat diet, resistance exercise)	12 weeks, Fortnightly contacts	*N* = 17 started *N* = 14 finished (82.3%)	*N* = 15 started *N* = 14 finished (93.3%)	61 ± 8	54 ± 13	29 ± 3	29 ± 3
Wei et al. (21)	Obesity and NAFLD	Ultrasound or magnetic resonance	Food intake (8 h): women 1,200–1,500 kcal/d and men 1,500–1800 kcal/d Fasting period (16 h): 0 kcal	women 1,200–1,500 kcal/d and men 1,500–1800 kcal/d	12 months, Fortnightly contacts	*N* = 45 started *N* = 34 finished (75.5%)	*N* = 43 started *N* = 40 finished (93.0%)	32 ± 10	31 ± 8	32 ± 3	32 ± 3
Cai et al. (22)	NAFLD	Ultrasound	Food intake (8 h): ad libitum intake, Fasting period (16 h): 0 kcal	20% ER	12 week, NA	*N* = 97 started *N* = 95 finished (97.9%)	*N* = 79 started *N* = 79 finished (100%)	33 ± 6	34 ± 6	26 ± 1	26 ± 2
WDF											
Lee et al. (23)	MASLD	Magnetic resonance	ER days: (550 kcal/d for women and 660 kcal/ d for men) Feast days: limit of 2000 kcal/d for women and 2,500 kcal/d for men	20% ER daily	12 week, Weekly calls	*N* = 32 started *N* = 29 finished (91.7%)	*N* = 31 started *N* = 26 finished (83.9%)	47(39–54)	51 (41–60)	27 (24–29)	27(24–30)
Sun et al. (16)	MASLD with abnormal glucose metabolism	Magnetic resonance	ER days: 500 kcal/d Feast days: ad libitum intake	25 kcal/kg	12 week, Fortnightly contacts	*N* = 30 started *N* = 28 finished (93.3%)	*N* = 30 started *N* = 28 finished (93.3%)	49 ± 10	46 ± 11	29 ± 3	31 ± 5
Wang et al. (24)	MAFLD	Ultrasound	ER days: (600 kcal/d for women and 800 kcal/ d for men) Feast days: women 1,400–1,600 kcal/d and men 1,600–1800 kcal/d	women 1,200–1,500 kcal/d and men 1,500–1800 kcal/d	12 week, Weekly calls	*N* = 30 started *N* = 27 finished(90.0%)	*N* = 30 started *N* = 27 finished(90.0%)	32.0 ± 7.0	29.0 ± 10.0	33.0 ± 5.0	32.0 ± 5.0
ADF											
Cai et al. (22)	NAFLD	Ultrasound	Fast day (24 h): 25% of baseline energy needs; feed day (24 h): ad libitum intake	80% of energy needs every day (20% ER)	12 week, NA	*N* = 95 started*N* = 90 finished (94.7%)	*N* = 79 started *N* = 79 finished (100%)	35.50 ± 4.417	34.54 ± 6.96	26.12 ± 2.21	26.34 ± 2.73

BMI, body mass index; CER, continuous energy restriction; IF, intermittent fasting; TRF, time-restricted feeding; WDF, 5:2 whole-day fasting; ADF, alternate-day fasting; ER, energy restriction; EI, energy intake; IQR, interquartile range; SD, standard deviation; SEM, standard error of the mean. Data are presented as mean ± SD unless otherwise indicated. Data for BMI are presented as median (IQR) in Karahan et al. (20) and Lee et al. (23), and age is presented as median (IQR) in Lee et al. (23). Completion rates were calculated based on the number of participants who started the intervention. Cai et al. (22) contributed two eligible intermittent fasting intervention arms, which were analyzed separately.

### Effects on hepatic fat content

In the pooled analysis, IF was associated with a greater reduction in CAP than CER (MD = −15.13 dB/m; 95% CI, −28.87 to −1.39; k = 5; *p* = 0.031; I^2^ = 28.2%; Supplementary Figure S1A). In subgroup analyses, no significant difference was observed between TRF and CER (MD = −10.37 dB/m; 95% CI, −33.80 to 13.06; k = 3; *p* = 0.386; I^2^ = 48.2%), whereas WDF showed a significant reduction in CAP relative to CER (MD = −21.43 dB/m; 95% CI, −36.63 to −6.24; k = 2; *p* = 0.006; I^2^ = 0%). However, no significant subgroup difference was detected (P for subgroup differences = 0.438). By contrast, MRI-PDFF did not differ significantly between IF and CER in the overall analysis (MD = −0.08%; 95% CI, −1.65 to 1.48; k = 4; *p* = 0.918; I^2^ = 56.6%; Supplementary Figure S1B). In subgroup analyses, neither TRF (MD = 0.75%; 95% CI, −0.08 to 1.58; k = 2; *p* = 0.075; I^2^ = 0%) nor WDF (MD = −1.91%; 95% CI, −4.60 to 0.78; k = 2; *p* = 0.164; I^2^ = 29.3%) showed a significant difference versus CER on MRI-PDFF. Likewise, no significant subgroup difference was observed (P for subgroup differences = 0.064).

### Effects on liver stiffness and transaminases

Changes in LSM did not differ significantly between IF and CER (MD = −0.41 kPa; 95% CI, −1.07 to 0.25; k = 9; *p* = 0.228; I^2^ = 75.8%; Supplementary Figure S2A). No significant subgroup difference was observed for LSM (P for subgroup differences = 0.792), and no significant subgroup effect was detected for TRF or WDF (*p* > 0.05). Overall, IF resulted in a modest but nonsignificant reduction in ALT compared with CER (MD = −3.23 U/L; 95% CI, −6.55 to 0.09; k = 8; *p* = 0.057; I^2^ = 0.0%; Supplementary Figure S2B). No overall difference was observed for AST (MD = −0.55 U/L; 95% CI, −3.14 to 2.04; k = 7; *p* = 0.676; I^2^ = 25.2%; Supplementary Figure S2C). In subgroup analyses, the WDF subgroup exhibited significantly greater reductions in both ALT (MD = −12.95 U/L; 95% CI, −22.42 to −3.47; k = 3; *p* = 0.007; I^2^ = 0%) and AST (MD = −5.52 U/L; 95% CI, −10.48 to −0.57; k = 3; *p* = 0.029; I^2^ = 0%) compared with CER. By contrast, the TRF subgroup showed no significant effect on ALT (MD = −1.87 U/L; 95% CI, −5.42 to 1.68; k = 5; *p* = 0.303; I^2^ = 0%) or AST (MD = 0.67 U/L; 95% CI, −1.14 to 2.49; k = 4; *p* = 0.467; I^2^ = 0%).

### Effects on body weight and BMI

Compared with CER, IF resulted in a greater reduction in body weight (MD = −1.29 kg; 95% CI, −1.98 to −0.61; k = 9; *p* < 0.001; I^2^ = 22.8%; Supplementary Figure S3A). In subgroup analyses, both TRF (MD = −1.31 kg; 95% CI, −2.42 to −0.20; k = 5; *p* = 0.021; I^2^ = 22.5%) and WDF (MD = −0.85 kg; 95% CI, −1.54 to −0.16; k = 4; *p* = 0.016; I^2^ = 0%) achieved significant reductions, whereas the single ADF trial reported a mean change in body weight of −2.87 kg (95% CI, −4.63 to −1.11). No significant subgroup difference was detected among IF subtypes for body weight (P for subgroup differences = 0.107). Similarly, IF significantly reduced BMI compared with CER (MD = −0.34 kg/m^2^; 95% CI, −0.55 to −0.13; k = 8; *p* = 0.002; I^2^ = 0.0%; Supplementary Figure S3B). TRF also significantly lowered BMI compared with CER (MD = −0.49 kg/m^2^; 95% CI, −0.83 to −0.15; k = 5; *p* = 0.005; I^2^ = 0%), whereas WDF and ADF showed no significant difference (*p* > 0.05). No significant subgroup difference was observed for BMI (P for subgroup differences = 0.543).

### Effects on glucose metabolism

Pooled analyses revealed no significant differences between IF and CER in fasting blood glucose (MD = 0.03 mmol/L; 95% CI, −0.15 to 0.21; k = 10; *p* = 0.727; I^2^ = 59.6%; Supplementary Figure S4A), fasting insulin (MD = 2.09 μIU/mL; 95% CI, −0.32 to 4.50; k = 5; *p* = 0.089; I^2^ = 48.1%; Supplementary Figure S4B), or HOMA-IR (MD = −0.15; 95% CI, −1.05 to 0.75; k = 7; *p* = 0.742; I^2^ = 81.7%; Supplementary Figure S4C). In subgroup analysis, the WDF subgroup was associated with a small increase in fasting blood glucose relative to CER; however, this finding should be interpreted cautiously given the overall null result, the limited number of studies, and the absence of a significant subgroup difference (P for subgroup differences = 0.427).

### Effects on lipid metabolism

Among lipid outcomes, only LDL-C demonstrated a modest reduction with IF compared with CER (MD = −0.08 mmol/L; 95% CI, −0.15 to −0.01; k = 10; *p* = 0.027; I^2^ = 0.0%; Supplementary Figure S5A). No significant differences were observed in TC (MD = −0.08 mmol/L; 95% CI, −0.39 to 0.22; k = 9; *p* = 0.602; I^2^ = 90.5%; Supplementary Figure S5B), TG (MD = 0.03 mmol/L; 95% CI, −0.05 to 0.11; k = 10; *p* = 0.452; I^2^ = 19.7%; Supplementary Figure S5C), or HDL-C (MD = 0.03 mmol/L; 95% CI, −0.16 to 0.21; k = 10; *p* = 0.783; I^2^ = 92.1%; Supplementary Figure S5D). No significant subgroup differences were observed for LDL-C (P for subgroup differences = 0.738), TC (*p* = 0.060), TG (*p* = 0.194), or HDL-C (*p* = 0.222).

### Subgroup analyses

Subgroup analyses were conducted to explore potential sources of heterogeneity. In the age-stratified analysis, among participants aged >43 years, IF was associated with higher fasting blood glucose relative to CER (MD = 0.16 mmol/L; 95% CI, 0.02 to 0.29; k = 5; *p* = 0.04; I^2^ = 0%; Supplementary Table S3). In the same age subgroup, heterogeneity in LSM disappeared, but no significant between-group difference was observed (MD = −0.14 kPa; 95% CI, −0.57 to 0.30; k = 4; *p* = 0.67; I^2^ = 0%; Supplementary Table S3). In the BMI-stratified analysis, among participants with BMI < 30 kg/m^2^, IF was associated with higher fasting blood glucose relative to CER (MD = 0.15 mmol/L; 95% CI, 0.03 to 0.27; k = 4; *p* = 0.01; I^2^ = 0%; Supplementary Table S4). Among participants with BMI ≥ 30 kg/m^2^, heterogeneity in MRI-PDFF disappeared, but no significant between-group difference was observed (MD = 0.88%; 95% CI, −0.18 to 1.94; k = 2; *p* = 0.10; I^2^ = 0%; Supplementary Table S4). Among Caucasian participants, heterogeneity in TC disappeared, but no significant difference between IF and CER was observed (MD = 0.20 mmol/L; 95% CI, −0.18 to 0.59; k = 3; *p* = 0.31; I^2^ = 0%; Supplementary Table S5). In studies without significant between-group differences in energy intake, heterogeneity disappeared for MRI-PDFF (MD = 0.70%; 95% CI, −0.11 to 1.50; k = 3; *p* = 0.09; I^2^ = 0%) and fasting blood glucose (MD = 0.23 mmol/L; 95% CI, −0.12 to 0.58; k = 2; *p* = 0.20; I^2^ = 0%; Supplementary Table S6). In studies with an intervention duration >13 weeks, heterogeneity in MRI-PDFF disappeared, although no significant between-group difference was observed (MD = 0.75%; 95% CI, −0.08 to 1.58; k = 2; *p* = 0.07; I^2^ = 0%; Supplementary Table S2). In imaging-modality subgroup analyses, magnetic resonance–based studies showed no significant between-group differences in LSM, fasting blood glucose, HOMA-IR, or TC, whereas IF significantly increased HDL-C compared with CER (MD = 0.08 mmol/L; 95% CI, 0.05 to 0.11; k = 4; *p* < 0.001; I^2^ = 0%; Supplementary Table S7). No significant differences were observed in ultrasound-based studies (Supplementary Table S7).

### Publication bias and risk of bias

Visual inspection of the funnel plots did not suggest marked asymmetry, and Egger’s regression tests were not statistically significant (*p* > 0.05). However, these assessments should be interpreted cautiously because the number of included studies for several outcomes was limited. According to the RoB 2 assessment, two studies (22.2%) were judged to be at low risk of bias, whereas the remaining seven (77.8%) raised some concerns (Supplementary Figure S7). The primary sources of bias were insufficient reporting of outcome assessor blinding and unclear handling of missing data. Due to the inherent nature of dietary interventions, blinding of participants was not feasible.

### Sensitivity analyses

Sensitivity analyses excluding one crossover trial (*n* = 32) and studies with sample sizes < 50 did not materially affect the overall direction or magnitude of the pooled estimates (Supplementary Table S8). However, after these exclusions, the between-group difference in CAP was no longer statistically significant (MD = −11.97; 95% CI, −31.08 to 7.14; k = 3; *p* = 0.22; I^2^ = 56.5%).

### Certainty of evidence

The certainty of evidence for the main outcomes, assessed using the GRADE approach, is summarized in Supplementary Table S9.

## Discussion

This systematic review and meta-analysis compared the effects of IF and CER on MASLD. Overall, the findings indicate that IF and CER have largely comparable effects, although some outcomes show modest advantages for IF. Specifically, IF was associated with greater reductions in body weight, BMI, and LDL-C levels. CAP-based analyses also suggested a potential benefit of IF for hepatic steatosis; however, this finding was not confirmed by MRI-PDFF and lost statistical significance in sensitivity analyses. Among different IF regimens, the most pronounced CAP reduction was observed with WDF, whereas TRF appeared to yield the greatest decreases in body weight and BMI. Although ADF produced the greatest weight loss in a single included study, the evidence remains limited. These findings suggest that different IF patterns may exert heterogeneous effects on metabolic outcomes, wheres the impact on hepatic steatosis remains uncertain and should be interpreted with caution. Sensitivity analyses confirmed the robustness of anthropometric and biochemical outcomes; however, the hepatic steatosis–related findings were less stable. In addition, several included trials had relatively small sample sizes, which may reduce the robustness of the pooled estimates and increase susceptibility to small-study effects, particularly for hepatic steatosis–related findings. For patients with MASLD who struggle with continuous calorie counting and long-term dietary adherence, IF may represent an alternative dietary strategy for selected individuals, particularly those who find continuous calorie restriction difficult to maintain.

Previous meta-analyses have investigated the effects of IF on hepatic and metabolic outcomes in various populations (15, 17, 26), and several studies have compared intermittent energy restriction (IER) with CER across metabolic disorders (27–29). However, evidence specifically focusing on patients with MASLD remains limited. The present analysis helps to address this gap by directly comparing IF and CER interventions in MASLD and further stratifying results by major IF modalities—TRF, ADF, and WDF—to offer a more comprehensive evaluation.

An important finding of this meta-analysis is the discordance between CAP and MRI-PDFF outcomes. CAP-based analyses suggested a potential benefit of IF compared with CER, whereas no significant difference was observed in MRI-PDFF. Notably, the apparent CAP advantage was no longer statistically significant in sensitivity analyses after excluding studies with small sample sizes, suggesting that this finding may not be robust and could have been influenced by small or high-impact studies. Moderate heterogeneity also persisted. Several factors may contribute to this discrepancy and the observed between-study heterogeneity. First, CAP and MRI-PDFF are based on distinct measurement principles. CAP quantifies ultrasound attenuation and is more susceptible to operator dependence, probe selection, and technical variability, and its performance may be influenced by patient characteristics such as body mass index (BMI) and the degree of hepatic steatosis (30). In individuals with obesity or more advanced steatosis, CAP measurements may be less reliable due to increased signal attenuation and technical limitations, whereas MRI-PDFF is less affected by these factors and maintains higher precision (31, 32). Second, with a limited number of included studies, pooled estimates may be highly sensitive to small-study effects or potential publication bias, which may contribute to both apparent significance and increased heterogeneity (33). Third, variations in baseline hepatic fat severity, intervention duration, dietary adherence, and concomitant weight loss may contribute to between-study variability and residual heterogeneity. Taken together, these factors likely contributed to the observed heterogeneity, which could not be fully explained despite subgroup analyses. In this context, MRI-based findings may provide a more reliable estimate of hepatic fat changes. Future trials should implement standardized imaging protocols incorporating both MRI-PDFF and CAP, with adequate sample sizes and individual-level data to facilitate more robust analyses. In subgroup analyses, WDF was associated with a greater reduction in CAP compared with TRF or ADF; however, these findings are based on a limited number of studies and should be interpreted as exploratory. This pattern may reflect the longer fasting duration in WDF, which could theoretically enhance metabolic switching, lipid mobilization, and hepatic fatty acid oxidation (34).

Our findings regarding body weight align with previous meta-analyses reporting greater weight loss with IF compared with CER in both healthy and overweight populations (27, 29, 35). Schwingshackl et al. (28) reported small but significant weight loss with IER in overweight and obese individuals, while Xu et al. (36) demonstrated that IF may be more effective than CER in improving body weight and metabolic syndrome parameters. In MASLD, where obesity and insulin resistance are key drivers, our results likewise suggest that IF may provide metabolic benefits compared with CER (37). Notably, the TRF subgroup in our analysis showed the largest pooled effect on body weight, whereas previous meta-analyses in healthy or obese adults often found ADF to be most effective (38, 39). This discrepancy may reflect differences in study design, population characteristics, and intervention duration. TRF may be easier for some patients to implement than more restrictive fasting regimens, which could partly explain its apparent advantage in this analysis; however, this interpretation remains speculative because adherence was not uniformly reported across trials. Conversely, ADF often induces greater short-term energy fluctuations but lower adherence, which could attenuate its effect in long-term trials. Therefore, the apparent advantage of TRF in our study may be partly attributable to better adherence rather than intrinsic superiority of the fasting pattern itself.

Although statistically significant, the magnitude of LDL-C reduction was modest and its clinical relevance remains uncertain. Mechanistically, fasting upregulates peroxisome proliferator-activated receptor *α* (PPARα) and its coactivator PGC-1α, which may enhance hepatic fatty acid oxidation and reduce triglyceride accumulation. This process suppresses very-low-density lipoprotein cholesterol (VLDL-C) synthesis, thereby lowering circulating LDL-C levels (28, 40). In contrast, no significant differences were observed between IF and CER for other metabolic or hepatic biochemical markers, suggesting that short-term metabolic improvements may be comparable between the two regimens (17, 29). Given that MASLD pathogenesis involves complex interactions among lipid metabolism, insulin sensitivity, and inflammation (41), more prolonged interventions may be necessary to reveal significant differences in these parameters.

IF is characterized by alternating periods of feeding and energy restriction, which induce metabolic switching between energy storage and utilization. During feeding, carbohydrates and lipids are mainly stored in the liver, skeletal muscle, and adipose tissue, whereas during fasting, the body depends on glycogenolysis and triglyceride mobilization to supply substrates for oxidative metabolism (42, 43). The potential hepatic effects of IF may be related to this transition between the fed and fasting states. This metabolic switching promotes the release and utilization of fatty acids and ketone bodies—signaling molecules that may regulate diverse cellular pathways, enhance stress resistance, improve organ function, and protect against metabolic and inflammatory diseases (12, 44, 45). Among the nutrient-sensing pathways that may contribute to these adaptive responses, the mechanistic target of rapamycin (mTOR) pathway represents one biologically plausible mediator. mTOR integrates signals from nutrients and insulin to regulate anabolic and catabolic processes relevant to metabolic homeostasis (46, 47). However, because the present study is based on comparative clinical meta-analytic data rather than mechanistic investigation, these interpretations should be regarded as biologically plausible rather than directly demonstrated by our pooled results. The differential effects of IF versus CER in MASLD are likely to involve multiple overlapping pathways and systemic factors, and further mechanistic studies are needed to clarify the contribution of specific signaling pathways.

The strengths of this study include its exclusive inclusion of randomized controlled trials (RCTs), stratified analyses across different IF regimens, and extensive sensitivity and subgroup analyses to explore heterogeneity. Nonetheless, several limitations should be acknowledged. First, the significance of CAP outcomes diminished after sensitivity testing, suggesting limited robustness. Second, residual heterogeneity persisted despite subgroup stratification, likely reflecting differences in intervention protocols, participant characteristics, and outcome assessment methods. Because of the limited number of studies within several subgroups, the ability to fully explore sources of heterogeneity was constrained. Third, most studies were of relatively short duration, which limited the assessment of long-term histological or safety outcomes. Finally, several included studies had relatively small sample sizes, which may have reduced the robustness of the pooled estimates and increased susceptibility to small-study effects. In particular, the loss of statistical significance for CAP after excluding studies with sample sizes < 50 suggests that this finding may not be stable and should be interpreted with caution. Future large-scale, well-controlled RCTs with standardized imaging and biochemical endpoints (e.g., MRI-PDFF, liver histology) and carefully matched energy intake are warranted to clarify the optimal IF approach for different patient subgroups defined by age, BMI, or metabolic comorbidities.

## Conclusion

In patients with MASLD, intermittent fasting was associated with greater reductions in body weight, BMI, and LDL-C than continuous energy restriction. CAP-based analyses suggested a possible benefit for hepatic steatosis, but this finding was not supported by MRI-PDFF and was no longer statistically significant in sensitivity analyses. Among intermittent fasting regimens, WDF appeared to show a greater reduction in CAP, whereas TRF appeared to be associated with greater reductions in body weight and BMI, although these subgroup findings should be interpreted cautiously. Further large-scale, long-term randomized trials are needed to confirm these findings.

## Data Availability

The original contributions presented in the study are included in the article/Supplementary material, further inquiries can be directed to the corresponding author/s.
