# Supplementary material for: Intermittent fasting versus continuous energy restriction in MASLD: a systematic review and meta-analysis
Source: Front Nutr. 2026 May 13;13:1833688. doi: 10.3389/fnut.2026.1833688 (PMC13213722; doi:10.3389/fnut.2026.1833688)

**SUPPLEMENTARY MATERIALS**

Intermittent Fasting vs Continuous Energy Restriction in MASLD: A Systematic Review and Meta-Analysis

**Table S1. Full search strategies used in PubMed, Embase, the Cochrane Central Register of Controlled Trials, and Web of Science.**

**PubMed search:**

| #1(Non-alcoholic Fatty Liver Disease[Mesh] OR (Non alcoholic Fatty Liver Disease[Title/Abstract]) OR (Fatty Liver, Nonalcoholic[Title/Abstract]) OR (Fatty Livers, Nonalcoholic[Title/Abstract]) OR (Liver, Nonalcoholic Fatty[Title/Abstract]) OR (Livers, Nonalcoholic Fatty[Title/Abstract]) OR (Nonalcoholic Fatty Liver[Title/Abstract]) OR (Nonalcoholic Fatty Livers[Title/Abstract]) OR (NAFLD[Title/Abstract]) OR (Nonalcoholic Fatty Liver Disease[Title/Abstract]) OR (Nonalcoholic Steatohepatitis[Title/Abstract]) OR (Nonalcoholic Steatohepatitides[Title/Abstract]) OR (Steatohepatitides, Nonalcoholic[Title/Abstract]) OR (Steatohepatitis, Nonalcoholic[Title/Abstract]) OR (Metabolic Dysfunction-Associated Fatty Liver Disease[Supplementary Concept]) OR (Metabolic Dysfunction-Associated Steatotic Liver Disease[Supplementary Concept]) OR (Metabolic dysfunction associated steatotic liver disease[Title/Abstract]) OR (NASH[Title/Abstract]) OR (MAFLD[Title/Abstract]) OR (MASLD[Title/Abstract]) |
| --- |
| #2(Intermittent Fasting[Title/Abstract]) OR (Fasting, Intermittent[Title/Abstract]) OR (Time Restricted Feeding[Title/Abstract]) OR (Feeding, Time Restricted[Title/Abstract]) OR (Time Restricted Feedings[Title/Abstract]) OR (Time Restricted Fasting[Title/Abstract]) OR (Fasting, Time Restricted[Title/Abstract]) OR (Restricted Fastings, Time[Title/Abstract]) OR (Time Restricted Eating [Title/Abstract]) OR (Eating, Time Restricted[Title/Abstract]) OR (Meal Skipping[Title/Abstract]) OR (Skipping, Meal[Title/Abstract]) OR (Breakfast Skipping[Title/Abstract]) OR (Skipping, Breakfast [Title/Abstract]) |
| #3(alternate-day fasting[Title/Abstract]) OR (ADF[Title/Abstract]) |
| #4(5:2 diet[Title/Abstract] )OR (5-2 diet[Title/Abstract] )OR (5:2 fasting[Title/Abstract]) |
| #5(whole-day fasting[Title/Abstract] )OR (whole day fasting[Title/Abstract]) |
| #6(fasting-mimicking diet[Title/Abstract] )OR (FMD[Title/Abstract]) |
| #7(Caloric Restrictionase[Title/Abstract]) OR (Restriction, Caloric[Title/Abstract]) OR (Caloric Restricted[Title/Abstract]) OR (Restricted, Caloric[Title/Abstract]) OR (Calorie Restricted Diet[Title/Abstract]) OR (Calorie Restricted Diets[Title/Abstract]) OR (Diet, Calorie Restricted[Title/Abstract]) OR (Restricted Diet, Calorie[Title/Abstract]) OR (Low-Calorie Diet [Title/Abstract]) OR (Diet, Low-Calorie[Title/Abstract]) OR (Low Calorie Diet[Title/Abstract]) OR (Low-Calorie Diets[Title/Abstract]) OR (energy restriction[Title/Abstract]) OR (reduced calorie[Title/Abstract]) |
| #8 #2 OR #3 OR #4 OR # 5 OR #6 OR #7 |
| #9 randomized controlled trial[Title/Abstract] |
| #10 controlled clinical trial[Title/Abstract] |
| #11 randomized[Title/Abstract] |
| #12 randomly[Title/Abstract] |
| #13 placebo[Title/Abstract] |
| #14 trial[Title/Abstract] |
| #15 groups[Title/Abstract] |
| #16 #9 OR #10 OR #11 OR #12 OR #13 OR #14 OR #15 |
| #17 #1 AND #8 AND #16 |
| #18 animals[mh] NOT humans[mh] |
| #19 #17 NOT #18 |

**Cochrane Central Register of Controlled Trials (CENTRAL) search:**

| #1( "non-alcoholic fatty liver disease" OR "non alcoholic fatty liver disease" OR "fatty liver, nonalcoholic" OR "fatty livers, nonalcoholic" OR "liver, nonalcoholic fatty" OR "livers, nonalcoholic fatty" OR "nonalcoholic fatty liver" OR "nonalcoholic fatty livers" OR NAFLD OR NASH OR MAFLD OR MASLD OR "nonalcoholic steatohepatitis" OR "nonalcoholic steatohepatitides" OR "steatohepatitis, nonalcoholic" OR "steatohepatitides, nonalcoholic" OR "metabolic dysfunction associated fatty liver disease" OR "metabolic dysfunction associated steatotic liver disease"):ti,ab,kw |
| --- |
| #2("intermittent fasting" OR "fasting, intermittent" OR "time restricted feeding" OR "feeding, time restricted" OR "time restricted feedings" OR "time restricted fasting" OR "fasting, time restricted" OR "restricted fastings, time" OR "time restricted eating" OR "eating, time restricted" OR "meal skipping" OR "skipping, meal" OR "breakfast skipping" OR "skipping, breakfast" OR "alternate-day fasting" OR ADF OR "5:2 diet" OR "5-2 diet" OR "5:2 fasting" OR "whole-day fasting" OR "whole day fasting" OR "fasting mimicking diet" OR FMD OR "caloric restriction" OR "restriction, caloric" OR "caloric restricted" OR "restricted, caloric" OR "calorie restricted diet" OR "calorie restricted diets" OR "diet, calorie restricted" OR "restricted diet, calorie" OR "low-calorie diet" OR "diet, low-calorie" OR "low calorie diet" OR "low-calorie diets" OR "energy restriction" OR "reduced calorie" OR "dietary energy restriction"):ti,ab,kw |
| #3 #1 AND #2 |

**Embase search:**

| #1**nonalcoholic fatty liver****'**/exp OR 'nonalcoholic fatty liver disease':ab,ti OR 'non alcoholic fatty liver disease':ab,ti OR 'fatty liver, nonalcoholic':ab,ti OR 'fatty livers, nonalcoholic':ab,ti OR 'liver, nonalcoholic fatty':ab,ti OR 'livers, nonalcoholic fatty':ab,ti OR 'nonalcoholic fatty liver':ab,ti OR 'nonalcoholic fatty livers':ab,ti OR nafld:ab,ti OR 'nonalcoholic steatohepatitis':ab,ti OR 'nonalcoholic steatohepatitides':ab,ti OR 'steatohepatitides, nonalcoholic':ab,ti OR 'steatohepatitis, nonalcoholic':ab,ti OR mafld:ab,ti OR masld:ab,ti OR nash:ab,ti OR 'metabolic dysfunction associated fatty liver disease':ab,ti OR 'metabolic dysfunction associated steatotic liver disease':ab,ti OR 'fatty liver'/exp OR 'steatohepatitis'/exp |
| --- |
| #2'intermittent fasting**'**/exp OR 'fasting, intermittent':ab,ti OR 'time restricted feeding':ab,ti OR 'feeding, time restricted':ab,ti OR 'time restricted feedings':ab,ti OR 'time restricted fasting':ab,ti OR 'fasting, time restricted':ab,ti OR 'restricted fastings, time':ab,ti OR 'time restricted eating':ab,ti OR 'eating, time restricted':ab,ti OR 'meal skipping':ab,ti OR 'skipping, meal':ab,ti OR 'breakfast skipping':ab,ti OR 'skipping, breakfast':ab,ti |
| #3'alternate day fasting':ab,ti OR adf:ab,ti |
| #4'5:2 diet':ab,ti OR '5-2 diet':ab,ti OR '5:2 fasting':ab,ti |
| #5'whole-day fasting':ab,ti OR 'whole day fasting':ab,ti |
| #6'fasting mimicking diet':ab,ti OR fmd:ab,ti |
| #7 'caloric restriction**'**/exp OR 'restriction, caloric':ab,ti OR 'caloric restricted':ab,ti OR 'restricted, caloric':ab,ti OR 'calorie restricted diet':ab,ti OR 'calorie restricted diets':ab,ti OR 'diet, calorie restricted':ab,ti OR 'restricted diet, calorie':ab,ti OR 'low-calorie diet':ab,ti OR 'diet, low-calorie':ab,ti OR 'low calorie diet':ab,ti OR 'low-calorie diets':ab,ti OR 'energy restriction':ab,ti OR 'reduced calorie':ab,ti OR 'dietary energy restriction':ab,ti |
| #8 #2 OR #3 OR #4 OR #5 OR #6 OR #7 |
| #9 'randomized controlled trial'/exp OR 'controlled clinical trial'/exp OR randomized:ab,ti OR randomly:ab,ti OR placebo:ab,ti OR trial:ab,ti OR groups:ab,ti |
| #10. #1 AND #8 AND #9 |
| #11 [animals]/lim NOT [humans]/lim |
| #12 #10 NOT #11 |

**Web of science search:**

| #1TS=("non-alcoholic fatty liver disease" OR "non alcoholic fatty liver disease" OR "fatty liver, nonalcoholic" OR "fatty livers, nonalcoholic" OR "liver, nonalcoholic fatty" OR "livers, nonalcoholic fatty" OR "nonalcoholic fatty liver" OR "nonalcoholic fatty livers" OR NAFLD OR NASH OR MAFLD OR MASLD OR "nonalcoholic steatohepatitis" OR "nonalcoholic steatohepatitides" OR "steatohepatitis, nonalcoholic" OR "steatohepatitides, nonalcoholic" OR "metabolic dysfunction associated fatty liver disease" OR "metabolic dysfunction associated steatotic liver disease") |
| --- |
| #2 TS=("intermittent fasting" OR "fasting, intermittent" OR "time restricted feeding" OR "feeding, time restricted" OR "time restricted feedings" OR "time restricted fasting" OR "fasting, time restricted" OR "restricted fastings, time" OR "time restricted eating" OR "eating, time restricted" OR "meal skipping" OR "skipping, meal" OR "breakfast skipping" OR "skipping, breakfast" OR "alternate-day fasting" OR ADF OR "5:2 diet" OR "5-2 diet" OR "5:2 fasting" OR "whole-day fasting" OR "whole day fasting" OR "fasting mimicking diet" OR FMD OR "caloric restriction" OR "restriction, caloric" OR "caloric restricted" OR "restricted, caloric" OR "calorie restricted diet" OR "calorie restricted diets" OR "diet, calorie restricted" OR "restricted diet, calorie" OR "low-calorie diet" OR "diet, low-calorie" OR "low calorie diet" OR "low-calorie diets" OR "energy restriction" OR "reduced calorie" OR "dietary energy restriction") |
| #3 TS=(randomized controlled trial OR controlled clinical trial OR randomized OR randomly OR placebo OR trial OR groups ) |
| #4 #1 AND #2 AND #3 |

**Table S2.** The effect of study duration on liver markers, blood lipids, and blood

glucose

| Outcome  variable | Stratification  variable | Number of  participants | Number of  studies | MD | 95%CI | P-value | *I2* |
| --- | --- | --- | --- | --- | --- | --- | --- |
| MRI-PDFF (%) | < 13 weeks | 123 | 2 | -1.91 | -4.60 to 0.78 | 0.16 | 29.3 |
|  | > 13 weeks | 208 | 2 | 0.75 | -0.08 to 1.58 | 0.07 | 0 |
| LSM (kPa) | < 13 weeks | 652 | 7 | -0.42 | -1.18 to 0.35 | 0.59 | 78.7 |
|  | > 13 weeks | 88 | 1 | -0.4 | -1.18 to 0.38 | 0.61 | NA |
| Fasting blood glucose (mmol/L) | < 13 weeks | 652 | 7 | 0.10 | -0.06 to 0.27 | 0.43 | 5 |
|  | > 13 weeks | 308 | 2 | -0.05 | -0.51  to 0.41 | 0.90 | 92.9 |
| HOMA-IR | < 13 weeks | 261 | 5 | 0.05 | -0.88 to 0.98 | 0.96 | 66 |
|  | > 13 weeks | 308 | 2 | -0.50 | -2.81 to 1.80 | 0.67 | 89.4 |
| TC (mmol/L) | < 13 weeks | 652 | 7 | -0.08 | -0.43 to 0.28 | 0.66 | 88.7 |
|  | > 13 weeks | 83 | 1 | -0.06 | -0.21 to 0.09 | 0.39 | NA |
| HDL-C (mmol/L) | < 13 weeks | 652 | 7 | 0.02 | -0.22 to 0.27 | 0.92 | 93.7 |
|  | > 13 weeks | 308 | 2 | 0.04 | -0.01 to 0.10 | 0.15 | 62.3 |

**Table S3.** The effect of age on liver markers, blood lipids, and blood glucose

| Outcome  variable | Stratification  variable | Number of  participants | Number of  studies | MD | 95%CI | P-value | *I2* |
| --- | --- | --- | --- | --- | --- | --- | --- |
| MRI-PDFF (%) | ≤ 43 years | 88 | 1 | 1 | -0.11 to 2.11 | 0.08 | NA |
|  | > 43 years | 343 | 3 | -0.75 | -2.95 to 1.44 | 0.59 | 58.9 |
| LSM (kPa) | ≤ 43 years | 537 | 4 | -0.61 | -1.79 to 0.56 | 0.39 | 87.8 |
|  | > 43 years | 213 | 4 | -0.14 | -0.57 to 0.3 | 0.67 | 0 |
| Fasting blood glucose (mmol/L) | ≤ 43 years | 537 | 4 | -0.02 | -0.29 to 0.25 | 092 | 71.2 |
|  | > 43 years | 423 | 5 | 0.16 | 0.02 to 0.29 | 0.04 | 0 |
| HOMA-IR | ≤ 43 years | 194 | 3 | 0.44 | -2.09 to 2.97 | 0.85 | 93.2 |
|  | > 43 years | 375 | 4 | -0.33 | -1.01 to 0.35 | 0.39 | 8.5 |
| TC (mmol/L) | ≤ 43 years | 203 | 4 | -0.03 | -0.31 to 0.26 | 0.92 | 56.1 |
|  | > 43 years | 573 | 4 | -0.17 | -0.73 to 0.40 | 0.59 | 91.8 |
| HDL-C (mmol/L) | ≤ 43 years | 423 | 5 | 0.02 | -0.40 to 0.44 | 0.96 | 96.1 |
|  | > 43 years | 537 | 5 | 0.06 | 0.00 to 0.11 | 0.07 | 52.1 |

**Table S4.** The effect of weight status on liver markers, blood lipids, and blood glucose

| Outcome  variable | Stratification  variable | Number of  participants | Number of  studies | MD | 95%CI | P-value | *I2* |
| --- | --- | --- | --- | --- | --- | --- | --- |
| MRI-PDFF (%) | BMI <30 kg/m² | 438 | 2 | -1.08 | -4.51 to 2.35 | 0.53 | 79.4 |
|  | BMI ≥ 30 kg/m2 | 148 | 2 | 0.88 | -0.18 to 1.94 | 0.10 | 0 |
| LSM (kPa) | BMI < 30kg/m2 | 406 | 3 | -0.86 | -2.38 to 0.65 | 0.26 | 84.6 |
|  | BMI ≥ 30 kg/m^2^ | 302 | 5 | -0.09 | -0.61 to 0.43 | 0.73 | 55.5 |
| Fasting blood glucose (mmol/L) | BMI < 30kg/m^2^ | 658 | 4 | 0.15 | 0.03 to 0.27 | 0.01 | 0 |
|  | BMI ≥ 30 kg/m^2^ | 254 | 5 | -0.04 | -0.40 to 0.31 | 0.82 | 69.1 |
| HOMA-IR | BMI < 30kg/m^2^ | 305 | 3 | -0.28 | -1.17 to 0.60 | 0.53 | 39 |
|  | BMI ≥ 30 kg/m^2^ | 506 | 4 | 0.09 | -1.59 to 1.77 | 0.92 | 89.8 |
| TC (mmol/L) | BMI < 30kg/m^2^ | 438 | 3 | -0.03 | -0.39 to 0.33 | 0.87 | 62.4 |
|  | BMI ≥ 30 kg/m^2^ | 302 | 5 | -0.10 | -0.60 to 0.39 | 0.69 | 94.2 |
| HDL-C (mmol/L) | BMI < 30kg/m^2^ | 658 | 4 | 0.02 | -0.04 to 0.08 | 0.51 | 16.7 |
|  | BMI ≥ 30 kg/m^2^ | 302 | 5 | 0.07 | -0.35 to 0.49 | 0.74 | 96.3 |

**Table S5.** Table S5. The effect of ethnicity on liver markers, blood lipids, and blood glucose

| Outcome  variable | Stratification  variable | Number of  participants | Number of  studies | MD | 95%CI | P-value | *I2* |
| --- | --- | --- | --- | --- | --- | --- | --- |
| LSM (kPa) | Asian | 608 | 5 | -0.66 | -1.48 to 0.16 | 0.11 | 76.5 |
|  | Caucasian | 132 | 3 | 0.28 | -0.58 to 1.14 | 0.52 | 36.2 |
| Fasting blood glucose (mmol/L) | Asian | 828 | 6 | 0.03 | -0.15 to 0.22 | 0.75 | 65 |
|  | Caucasian | 132 | 3 | -0.16 | -1.23 to 0.90 | 0.77 | 61.1 |
| HOMA-IR | Asian | 485 | 5 | -0.24 | -1.30 to 0.82 | 0.65 | 86.1 |
|  | Caucasian | 84 | 2 | 0.91 | -2.91 to 4.74 | 0.64 | 75.1 |
| TC (mmol/L) | Asian | 608 | 5 | -0.19 | -0.56 to 0.18 | 0.31 | 93.3 |
|  | Caucasian | 132 | 3 | 0.20 | -0.18 to 0.59 | 0.31 | 0 |
| HDL-C (mmol/L) | Asian | 828 | 6 | 0.10 | -0.05 to 0.25 | 0.19 | 80.5 |
|  | Caucasian | 132 | 3 | -0.19 | -0.59 to 0.21 | 0.35 | 95.8 |

Table S6. The effect of between-group differences in energy intake on liver markers, blood lipids, and blood glucose

| Outcome  variable | Stratification  variable | Number of  participants | Number of  studies | MD | 95%CI | P-value | I2 |
| --- | --- | --- | --- | --- | --- | --- | --- |
| MRI-PDFF (%) | EI sig. | 63 | 1 | -3.10 | -5.99 to 0.21 | 0.05 | NA |
|  | EI n.s. | 368 | 3 | 0.70 | -0.11 to 1.50 | 0.09 | 0 |
| LSM (kPa) | EI sig. | 107 | 2 | -0.31 | -0.87 to 0.25 | 0.28 | 4.6 |
|  | EI n.s. | 623 | 6 | -0.43 | -1.32 to 0.46 | 0.34 | 81.2 |
| Fasting blood glucose (mmol/L) | EI sig. | 843 | 7 | 0 | -0.20 to 0.20 | 1.00 | 65.3 |
|  | EI n.s. | 515 | 2 | 0.23 | -0.12  to 0.58 | 0.20 | 0 |
| HOMA-IR | EI sig. | 117 | 2 | 0.19 | -1.49  to 1.87 | 0.82 | 75.7 |
|  | EI n.s. | 452 | 5 | -0.30 | -1.42  to 0.82 | 0.60 | 74.9 |
| TC (mmol/L) | EI sig. | 54 | 1 | 0.22 | -0.11  to 0.55 | 0.19 | NA |
|  | EI n.s. | 515 | 6 | -0.16 | -0.52  to 0.20 | 0.38 | 91.7 |
| HDL-C (mmol/L) | EI sig. | 117 | 2 | 0.42 | -0.27 to 1.12 | 0.24 | 93.4 |
|  | EI n.s. | 843 | 7 | -0.06 | -0.21 to 0.10 | 0.45 | 95.1 |

Abbreviations: EI sig., significant between-group difference in energy intake; EI n.s., no significant between-group difference in energy intake.

**Table S7.** Table S7. The effect of imaging modality on liver markers, blood lipids, and blood glucose

| Outcome  variable | Stratification  variable | Number of  participants | Number of  studies | MD | 95%CI | P-value | *I2* |
| --- | --- | --- | --- | --- | --- | --- | --- |
| LSM(kPa) | magnetic resonance | 211 | 3 | -0.19 | -0.61 to 0.23 | 0.37 | 0 |
|  | ultrasound | 529 | 5 | -0.56 | -1.63 to 0.52 | 0.30 | 84.7 |
| Fasting blood glucose (mmol/L) | magnetic resonance | 431 | 4 | -0.07 | -0.35 to 0.22 | 0.64 | 79.1 |
|  | ultrasound | 529 | 5 | 0.15 | -0.04 to 0.33 | 0.12 | 17.6 |
| HOMA-IR | magnetic resonance | 431 | 4 | -0.62 | -1.70 to 0.45 | 0.25 | 71.0 |
|  | ultrasound | 138 | 3 | 0.60 | -1.07 to 2.27 | 0.48 | 78.7 |
| TC (mmol/L) | magnetic resonance | 529 | 5 | -0.25 | -0.91 to 0.40 | 0.45 | 96.9 |
|  | ultrasound | 211 | 3 | 0.02 | -0.29 to 0.32 | 0.92 | 48.3 |
| HDL-C (mmol/L) | magnetic resonance | 531 | 4 | 0.08 | 0.05 to 0.11 | <0.001 | 0 |
|  | ultrasound | 529 | 5 | 0.00 | -0.34 to 0.34 | 1.00 | 93.8 |

**Table S8.** Results of sensitivity analyses excluding crossover and small-sample (<50 participants) trials

| Outcome variable | Number of studies | MD | 95%CI | *I2* |
| --- | --- | --- | --- | --- |
| CAP (dB/m) | 3 | -11.97 | -31.08 to 7.14 | 56.5 |
| MRI-PDFF (%) | 4 | -0.08 | -1.65 to 1.48 | 56.6 |
| LSM (kPa) | 6 | -0.45 | -1.25 to 0.35 | 81.8 |
| ALT (U/L) | 6 | -3.65 | -8.08 to 0.78 | 15.8 |
| AST (U/L) | 6 | -0.89 | -3.66 to 1.88 | 28.3 |
| Body weight (kg) | 7 | -1.23 | -2.02 to -0.45 | 30.7 |
| BMI (kg/㎡) | 5 | -0.27 | -0.51 to -0.04 | 0 |
| Fasting blood glucose (mmol/L) | 7 | 0.01 | -0.18 to 0.19 | 65.9 |
| Fasting insulin (µIU/mL) | 4 | 2.76 | -1.66 to 7.18 | 58.9 |
| HOMA-IR | 5 | -0.01 | -1.10 to 1.07 | 84.7 |
| LDL-C (mmol/L) | 7 | -0.08 | -0.15 to -0.01 | 14.2 |
| TC (mmol/L) | 6 | -0.11 | -0.47 to 0.26 | 92.5 |
| TG (mmol/L) | 7 | 0.02 | -0.08 to 0.11 | 32.6 |
| HDL-C (mmol/L) | 7 | 0.03 | -0.21 to 0.27 | 93.8 |

**Table S9.** GRADE assessment of the certainty of evidence for the main outcomes

| **Outcome** | **Pooled effect (IF vs. CER)** | **Studies (k)** | **Heterogeneity (I²)** | **Certainty of evidence** | **Main reasons for downgrading** |
| --- | --- | --- | --- | --- | --- |
| Body weight | MD = −1.29 kg (95% CI, −1.98 to −0.61) | 9 | 22.8% | Moderate | Risk of bias |
| BMI | MD = −0.34 kg/m² (95% CI, −0.55 to −0.13) | 8 | 0.0% | Moderate | Risk of bias |
| CAP | MD = −15.13 dB/m (95% CI, −28.87 to −1.39) | 5 | 28.2% | Low | Risk of bias; imprecision |
| MRI-PDFF | MD = −0.08% (95% CI, −1.65 to 1.48) | 4 | 56.6% | Low | Risk of bias; inconsistency |
| LSM | MD = −0.41 kPa (95% CI, −1.07 to 0.25) | 9 | 75.8% | Low | Risk of bias; inconsistency |
| ALT | MD = −3.23 U/L (95% CI, −6.55 to 0.09) | 8 | 0.0% | Moderate | Risk of bias |
| AST | MD = −0.55 U/L (95% CI, −3.14 to 2.04) | 7 | 25.2% | Low | Risk of bias; imprecision |
| LDL-C | MD = −0.08 mmol/L (95% CI, −0.15 to −0.01) | 10 | 0.0% | Moderate | Risk of bias |
| TC | MD = −0.08 mmol/L (95% CI, −0.39 to 0.22) | 9 | 90.5% | Very low | Risk of bias; inconsistency; imprecision |
| TG | MD = 0.03 mmol/L (95% CI, −0.05 to 0.11) | 10 | 19.7% | Moderate | Risk of bias |
| HDL-C | MD = 0.03 mmol/L (95% CI, −0.16 to 0.21) | 10 | 92.1% | Very low | Risk of bias; inconsistency; imprecision |
| Fasting blood glucose | MD = 0.03 mmol/L (95% CI, −0.15 to 0.21) | 10 | 59.6% | Low | Risk of bias; inconsistency |
| Fasting insulin | MD = 2.09 µIU/mL (95% CI, −0.32 to 4.50) | 5 | 48.1% | Low | Risk of bias; imprecision |
| HOMA-IR | MD = −0.15 (95% CI, −1.05 to 0.75) | 7 | 81.7% | Very low | Risk of bias; inconsistency; imprecision |

*Abbreviations: GRADE, Grading of Recommendations Assessment, Development and Evaluation; IF, intermittent fasting; CER, continuous energy restriction; MD, mean difference; CI, confidence interval; CAP, controlled attenuation parameter; MRI-PDFF, magnetic resonance imaging–proton density fat fraction; LSM, liver stiffness measurement; ALT, alanine aminotransferase; AST, aspartate aminotransferase; LDL-C, low-density lipoprotein cholesterol; TC, total cholesterol; TG, triglycerides; HDL-C, high-density lipoprotein cholesterol; HOMA-IR, homeostasis model assessment of insulin resistance.*

*Footnote:* Evidence from randomized controlled trials was initially rated as high certainty and downgraded, where appropriate, for risk of bias, inconsistency, imprecision, indirectness, or publication bias.

**Supplementary Figure1**. Forest plot of randomized controlled trials comparing intermittent fasting (IF) with continuous energy restriction (CER) on changes in (A) controlled attenuation parameter (CAP) and (B) magnetic resonance imaging–proton density fat fraction (MRI-PDFF). Effect sizes are expressed as mean differences (MDs) with 95% confidence intervals (CIs), pooled using a random-effects model. Grey squares represent individual study effect estimates, with square size proportional to study weight; horizontal lines indicate 95% CIs; and diamond shapes represent pooled effect estimates. Prediction intervals are shown for the pooled estimates. Subgroup pooled estimates and p values for subgroup effects are presented where applicable. Between-study heterogeneity was assessed using the I² statistic. Abbreviations: IF, intermittent fasting; CER, continuous energy restriction; TRF, time-restricted feeding; WDF, whole-day fasting; ADF, alternate-day fasting.


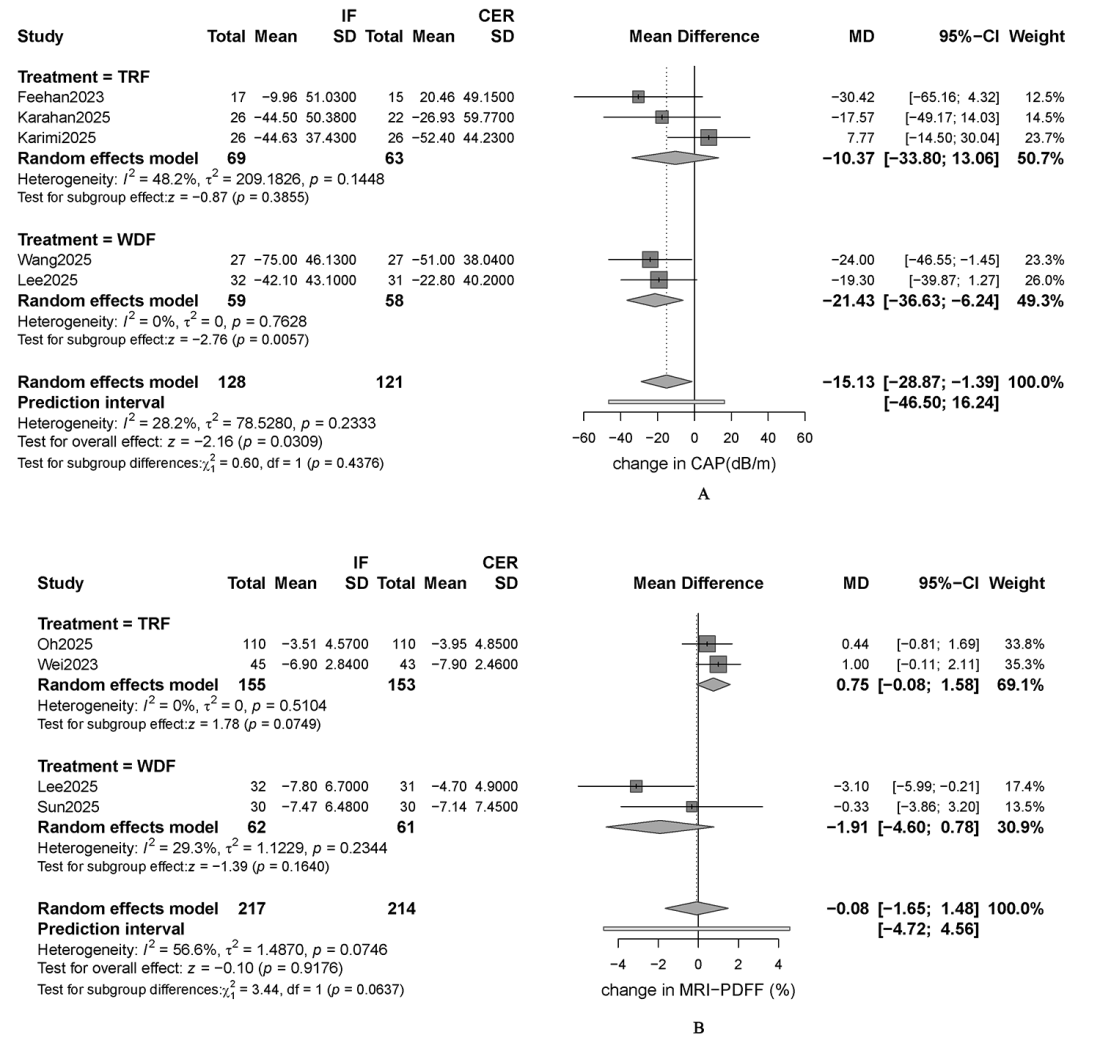


**Supplementary Figure2**. Forest plot of randomized controlled trials comparing IF with CER on changes in (A) liver stiffness measurement (LSM), (B) alanine aminotransferase (ALT), and (C) aspartate aminotransferase (AST). Effect sizes are expressed as MDs with 95% CIs using a random-effects model. Grey squares represent individual study effect estimates, with square size proportional to study weight; horizontal lines indicate 95% CIs; and diamond shapes represent pooled effect estimates. Prediction intervals are shown for the pooled estimates. Subgroup pooled estimates and p values for subgroup effects are presented where applicable. Between-study heterogeneity was assessed using the I² statistic. Abbreviations: IF, intermittent fasting; CER, continuous energy restriction; TRF, time-restricted feeding; WDF, whole-day fasting; ADF, alternate-day fasting.


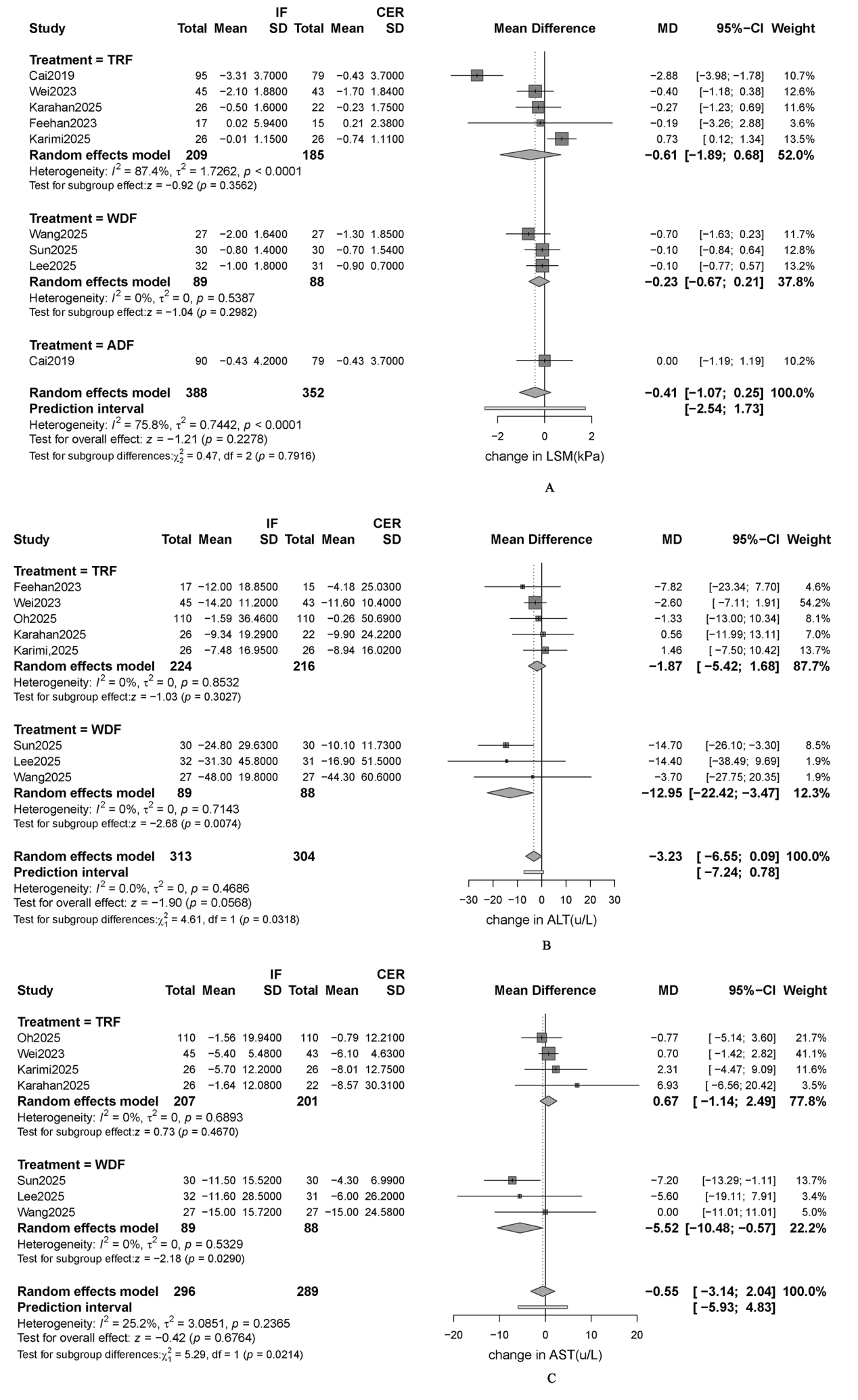


**Supplementary Figure3**. Forest plot of randomized controlled trials comparing IF with CER on changes in (A) body weight and (B) body mass index (BMI). Effect sizes are expressed as MDs with 95% CIs using a random-effects model. Grey squares represent individual study effect estimates, with square size proportional to study weight; horizontal lines indicate 95% CIs; and diamond shapes represent pooled effect estimates. Prediction intervals are shown for the pooled estimates. Subgroup pooled estimates and p values for subgroup effects are presented where applicable. Between-study heterogeneity was assessed using the I² statistic. Abbreviations: IF, intermittent fasting; CER, continuous energy restriction; TRF, time-restricted feeding; WDF, whole-day fasting; ADF, alternate-day fasting.


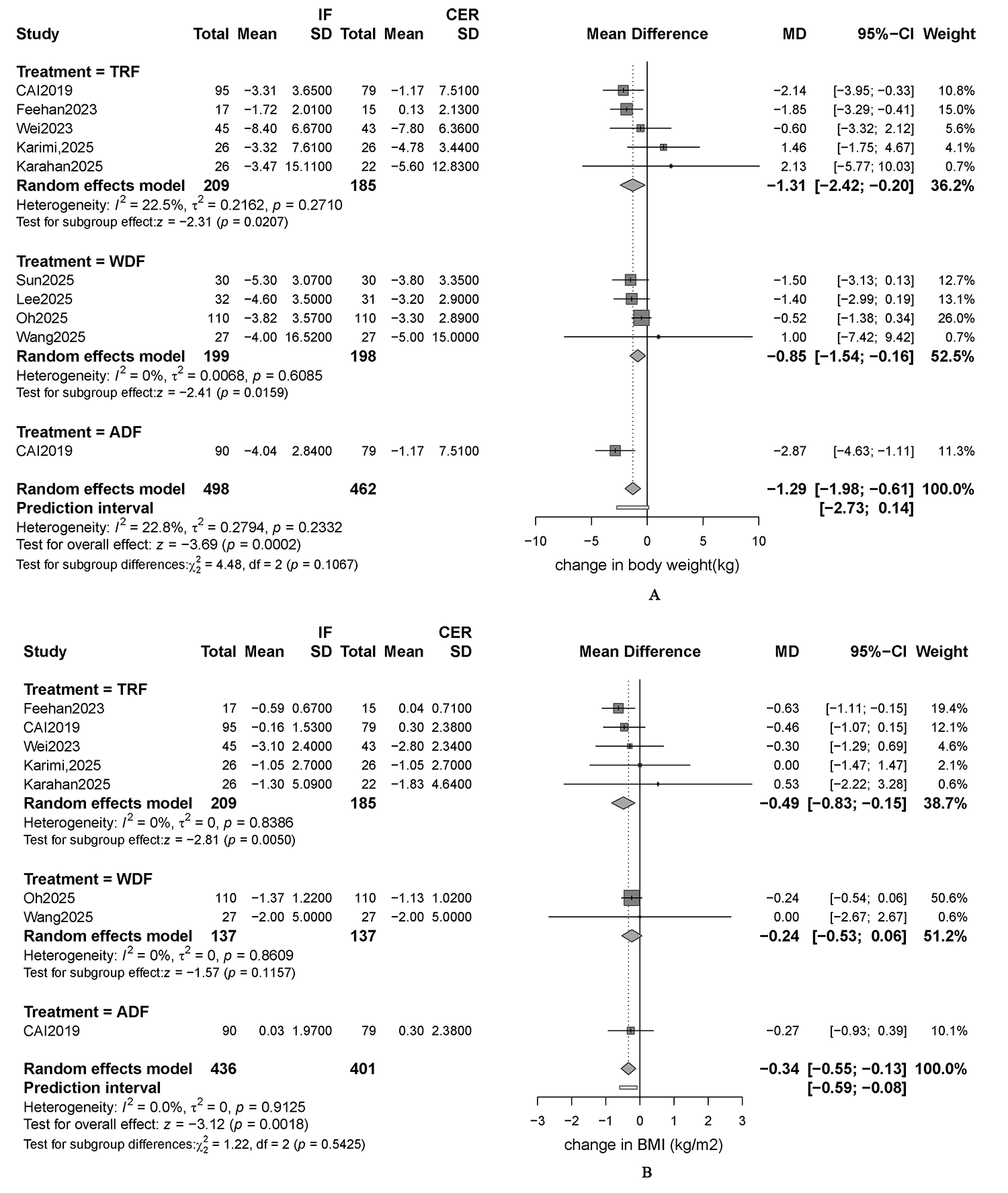


**Supplementary Figure4**. Forest plot of randomized controlled trials comparing IF with CER on changes in (A) fasting blood glucose, (B) fasting insulin, and (C) homeostasis model assessment of insulin resistance (HOMA-IR). Effect sizes are expressed as MDs with 95% CIs using a random-effects model. Grey squares represent individual study effect estimates, with square size proportional to study weight; horizontal lines indicate 95% CIs; and diamond shapes represent pooled effect estimates. Prediction intervals are shown for the pooled estimates. Subgroup pooled estimates and p values for subgroup effects are presented where applicable. Between-study heterogeneity was assessed using the I² statistic. Abbreviations: IF, intermittent fasting; CER, continuous energy restriction; TRF, time-restricted feeding; WDF, whole-day fasting; ADF, alternate-day fasting.


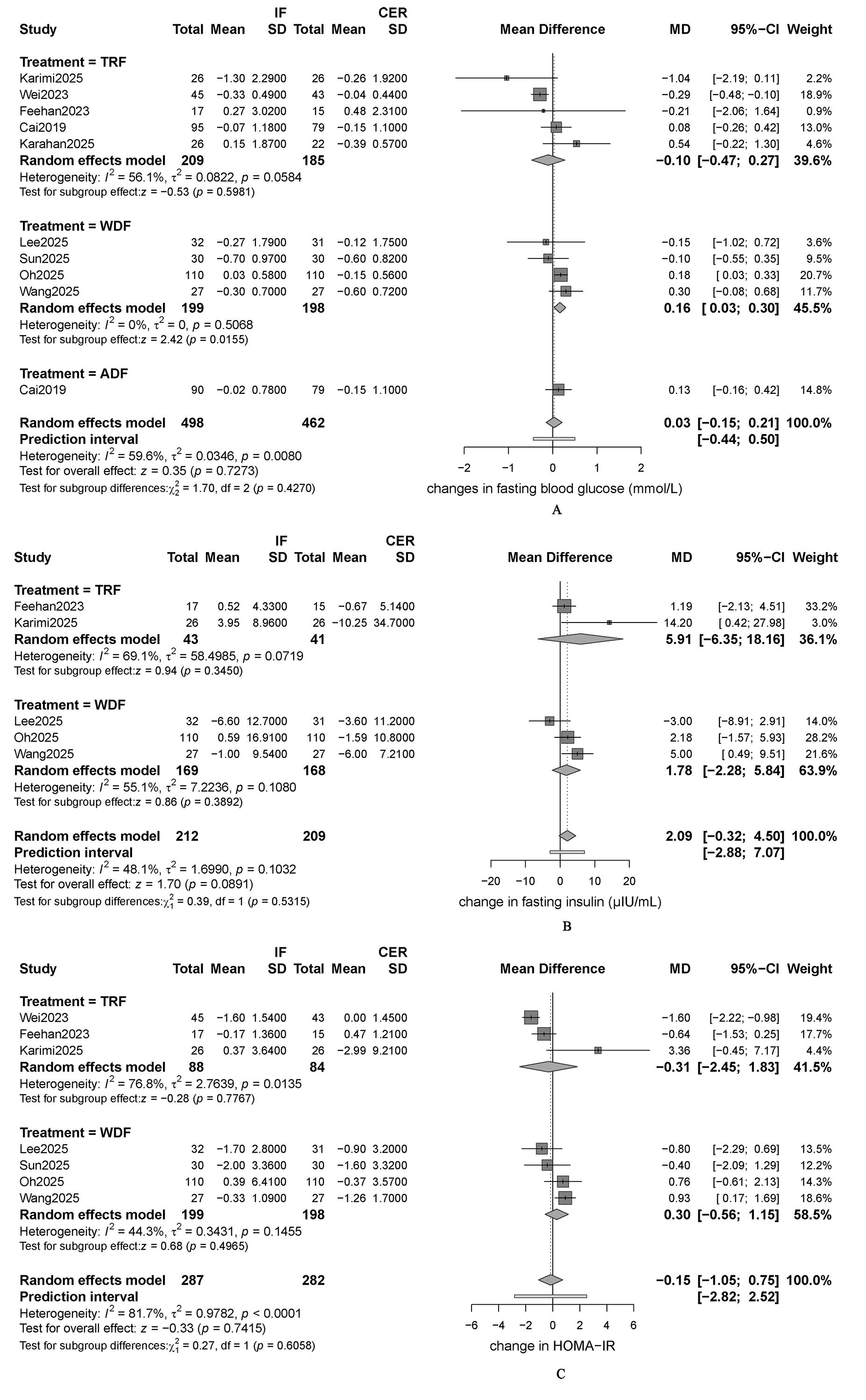


**Supplementary Figure5**. Forest plot of randomized controlled trials comparing IF with CER on changes in (A) low-density lipoprotein cholesterol (LDL-C), (B) total cholesterol (TC), (C) triglycerides (TG), and (D) high-density lipoprotein cholesterol (HDL-C). Effect sizes are expressed as MDs with 95% CIs using a random-effects model. Grey squares represent individual study effect estimates, with square size proportional to study weight; horizontal lines indicate 95% CIs; and diamond shapes represent pooled effect estimates. Prediction intervals are shown for the pooled estimates. Subgroup pooled estimates and p values for subgroup effects are presented where applicable. Between-study heterogeneity was assessed using the I² statistic. Abbreviations: IF, intermittent fasting; CER, continuous energy restriction; TRF, time-restricted feeding; WDF, whole-day fasting; ADF, alternate-day fasting.


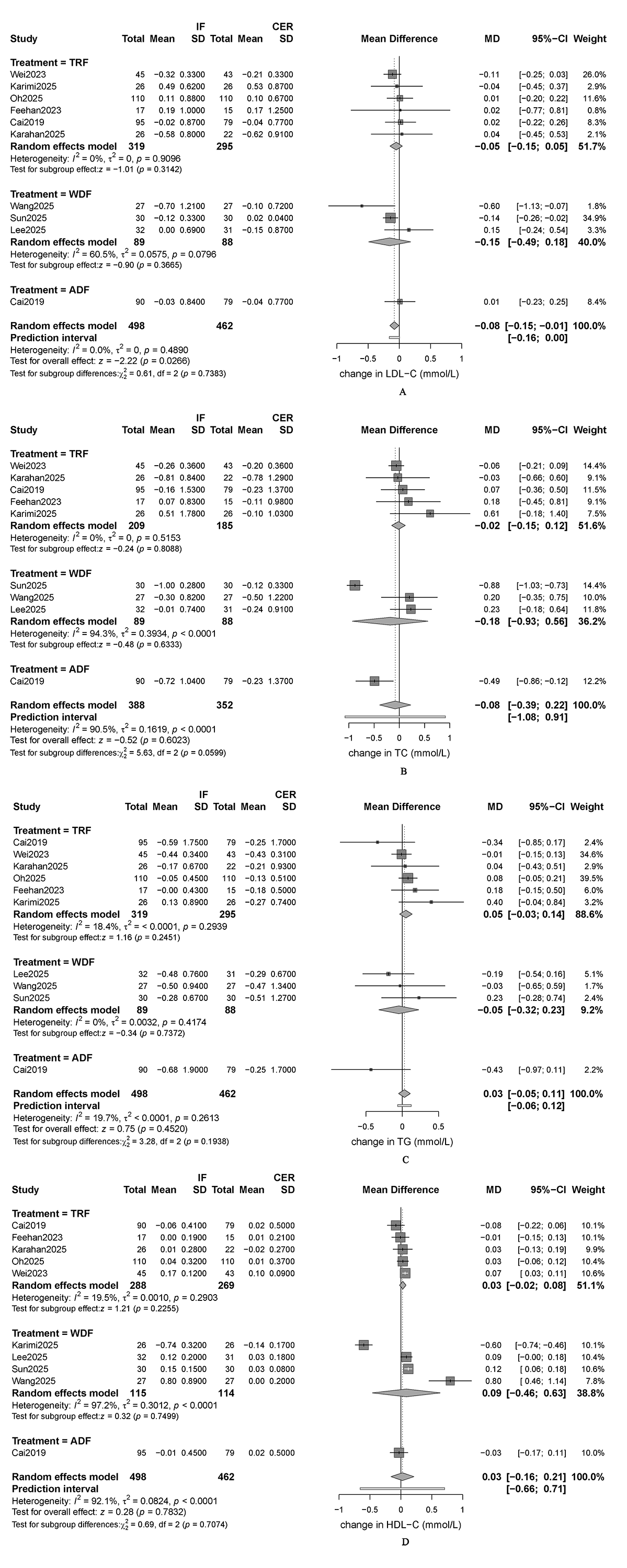


**Supplementary Figure6**. Funnel plots assessing potential publication bias for the effects of intermittent fasting (IF) versus continuous energy restriction (CER) on changes in (A) CAP, (B) MRI-PDFF, (C) LSM, (D) ALT, (E) AST, (F) body weight, (G) BMI, (H) fasting blood glucose, (I) fasting insulin, (J) HOMA-IR, (K) low-density lipoprotein cholesterol (LDL-C), (L) total cholesterol (TC), (M) triglycerides (TG), and (N) high-density lipoprotein cholesterol (HDL-C).

Abbreviations: IF, intermittent fasting; CER, continuous energy restriction; CAP, controlled attenuation parameter; MRI-PDFF, magnetic resonance imaging–proton density fat fraction; LSM, liver stiffness measurement; ALT, alanine aminotransferase; AST, aspartate aminotransferase; BMI, body mass index; HOMA-IR, homeostasis model assessment of insulin resistance; LDL-C, low-density lipoprotein cholesterol; TC, total cholesterol; TG, triglycerides; HDL-C, high-density lipoprotein cholesterol.
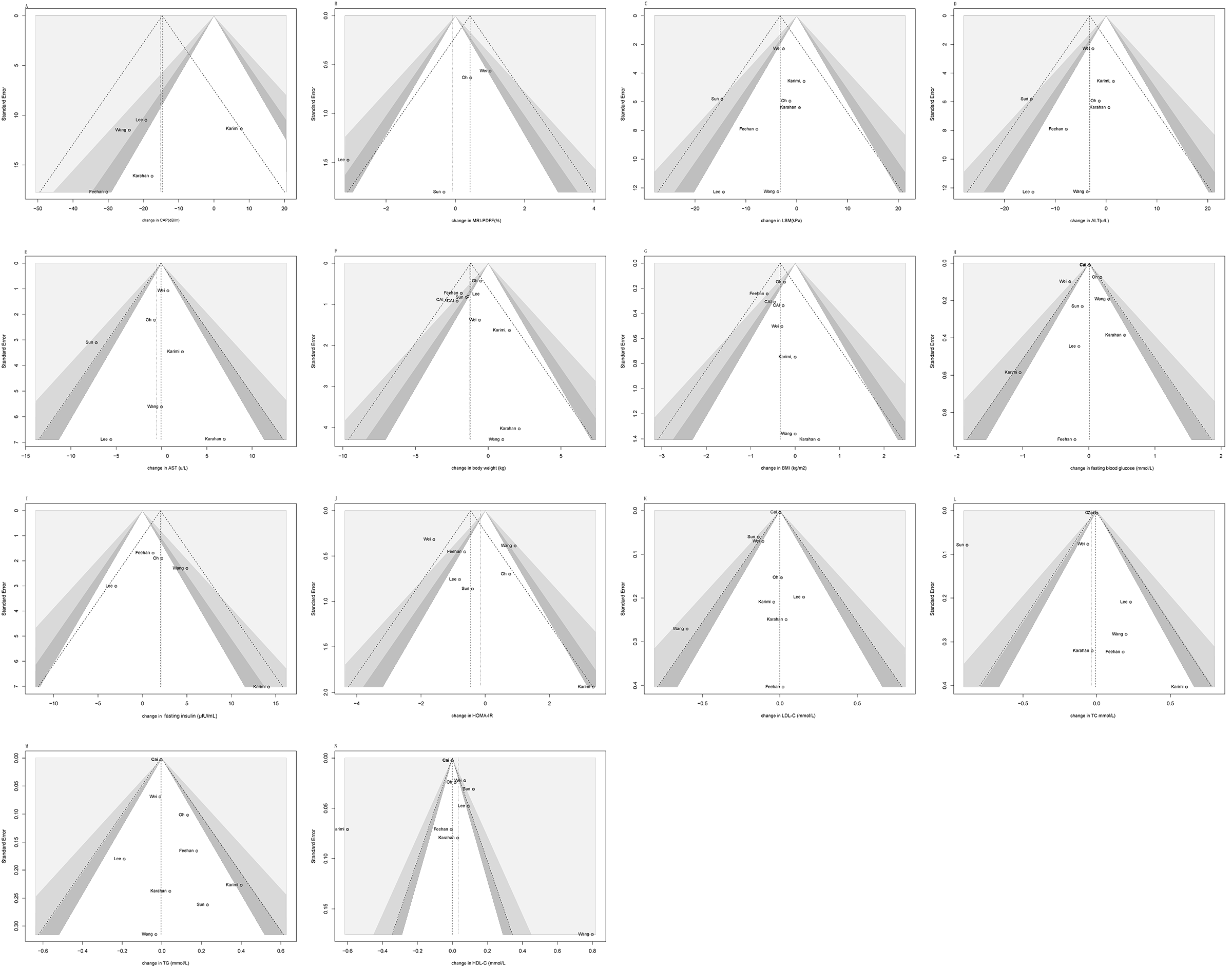


**Supplementary Figure7**. Risk-of-bias assessment of the included randomized controlled trials using the Cochrane Risk of Bias 2 (RoB 2) tool. Judgements were classified as low risk, some concerns, or high risk.


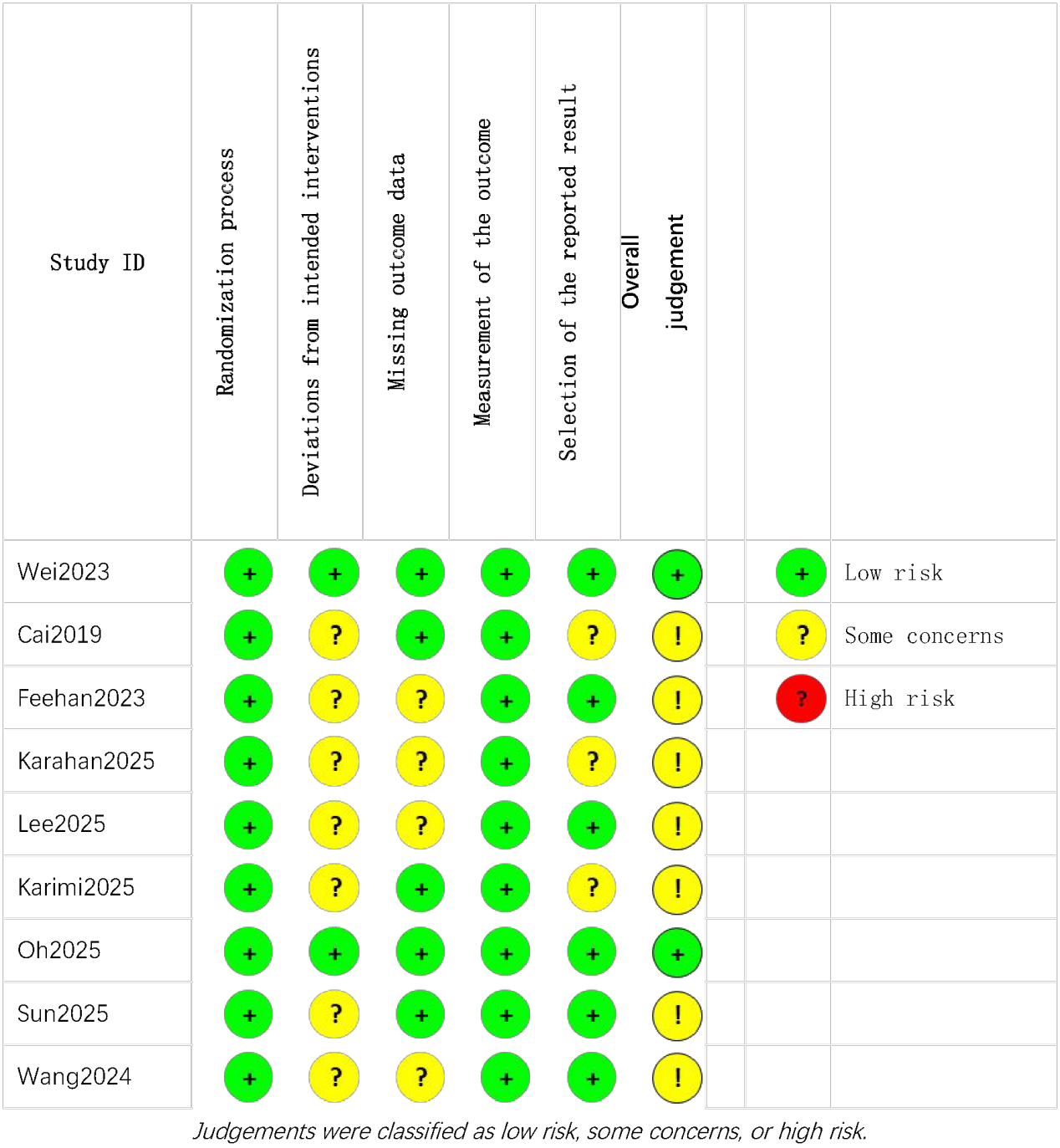

Supplement: Supplementary file 1 [file Supplementary_file_1.docx]
